# Micropropagation of *Chaenomeles japonica*: A Step towards Production of Polyphenol-rich Extracts Showing Antioxidant and Antimicrobial Activities

**DOI:** 10.3390/molecules24071314

**Published:** 2019-04-03

**Authors:** Małgorzata Kikowska, Agata Włodarczyk, Monika Rewers, Elwira Sliwinska, Elżbieta Studzińska-Sroka, Ewa Witkowska-Banaszczak, Anna Stochmal, Jerzy Żuchowski, Jolanta Dlugaszewska, Barbara Thiem

**Affiliations:** 1 Department of Pharmaceutical Botany and Plant Biotechnology, Poznan University of Medical Sciences, 61-861 Poznań, Poland; agata_nahorska@wp.pl (A.W.); bthiem@ump.edu.pl (B.T.); 2 Laboratory of Molecular Biology and Cytometry, Department of Agricultural Biotechnology, UTP University of Science and Technology, 85-789 Bydgoszcz, Poland; mrewers@utp.edu.pl (M.R.); elwira@utp.edu.pl (E.S.); 3 Department of Pharmacognosy, Poznan University of Medical Sciences, 60-781 Poznań, Poland; elastudzinska@ump.edu.pl (E.S.-S.); banewit@wp.pl (E.W.-B.); 4 Department of Biochemistry and Crop Quality, Institute of Soil Science and Plant Cultivation, State Research Institute, 24-100 Puławy, Poland; asf@iung.pulawy.pl (A.S.); jzuchowski@iung.pulawy.pl (J.Ż.); 5 Department of Genetics and Pharmaceutical Microbiology, Poznan University of Medical Sciences, 4 Święcickiego St., 60-781 Poznań, Poland; jdlugasz@ump.edu.pl

**Keywords:** antimicrobial activity, antioxidant tests, DNA content, Japanese quince, micropropagation, polyphenols

## Abstract

A protocol for *C. japonica* micropropagation with a confirmation of genome size stability of the in vitro-propagated plantlets was developed. The highest number of shoots multiplied in vitro was obtained on Murashige & Skoog medium (MS) with 1.0 mg L^−1^ N6-benzyladenine plus 1.0 mg L^−1^ indole-3-acetic acid. The highest number of roots was observed for the shoots on MS with 15 g L^−1^ sucrose plus 1.0 mg L^−1^ indole-3-acetic acid. The acclimatization rate was significantly high. The qualitative HPLC analyses confirmed the presence of phenolic acids and flavonoids in the extracts. The extracts from both shoot cultures and the leaves from field-grown plants revealed antioxidant activity and they exhibited moderate antimicrobial activity. The conducted research confirmed the regeneration potential of genetically-stable plants of *C. japonica* under in vitro conditions, the ability of the plantlets to produce polyphenols as those present in field-grown plants, as well as their antioxidant potential.

## 1. Introduction

*Chaenomeles japonica* is a species that belongs to the genus of Chaenomeles Lindl. (the Maloideae subfamily, the Rosaceae family); the Chaenomeles comprises five species [1,2]. *C. japonica*, which is a dwarf shrub growing up to 1.2 m, is present in Japan and it usually overgrows hillsides, riverbanks, and lakeshores [1]. In Europe, the plant was introduced in 1869 and domesticated in the northern countries as a fruit crop [3].

The content of vitamin C, organic acids, phenolics, fiber, pectin, and sugars in the fruits indicates their use in the food industry [4,5,6,7,8,9,10]. The fruits of the genus Chaenomeles (Mugua) have been used in Chinese medicine for arthritis, hepatitis, asthma, diarrhea, and for a common cold [11,12], while the fruits of *C. japonica* have been used as an astringent and in stomach diseases [13].

Phytochemical studies that were performed on *C. japonica* and summarized by Xu et al. [14] yielded secondary metabolites: in fruits—roseoside, monoterpene glucosides, epicatechin, and leucoanthocyanin; in leaves—flavonol glycosides and epicatechin; and, in roots—daucosterol, ursolic, oleanolic acid, pomolic acid, prunasin, and epicatechin [14].

Due to the extreme heterogeneity of plants that are cross-pollinated and propagated by seeds, no varieties were available for this species for many years [3,15]. The improvement in breeding and selection created the large genetic diversity in Chaenomeles species, which resulted in the occurrence of several selected genotypes of *C. japonica* in Europe. Although Chaenomeles can be easily propagated generatively, the selected genotypes should be vegetatively propagated, because of their unique qualities [16].

Only a few scientific papers on micropropagation of *C. japonica* have been developed [17,18,19], but the results have proven to be unsatisfactory and incomplete. The efficient and rapid plant propagation in in vitro cultures under optimized conditions is still in demand in regards to obtaining the biomass for the phytochemical and biological analyses. There are not many research papers on the phytochemical and biological activity of extracts from leaves, so this work enriches the knowledge regarding the profile of the compounds that are present in both leaves of wild plants and in vitro shoot culture.

The aim of the work was to develop a reproducible protocol for *C. japonica* clonal propagation through axillary branching with the confirmation of (i) genome size stability of in vitro-propagated plantlets, (ii) maintenance of their ability to produce selected polyphenols under in vitro conditions, (iii) antioxidant activity, and (iv) antimicrobial activity.

## 2. Results and Discussion

**Figure 1:**
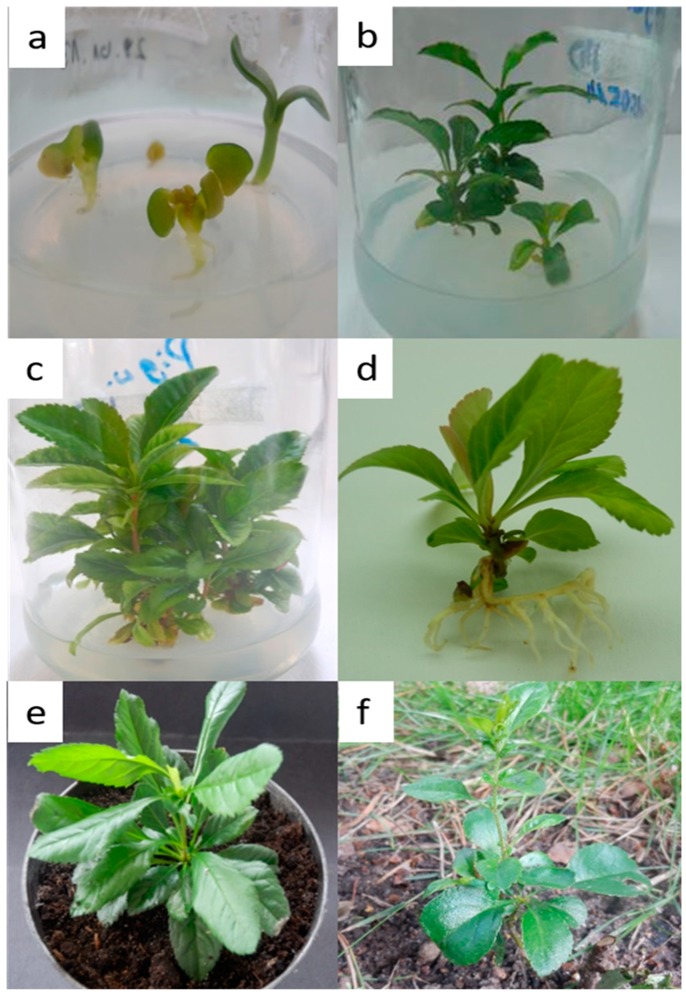
In vitro propagation of *Chaenomeles japonica*: (**a**) aseptic seedlings; (**b**) shoot tip explants; (**c**) in vitro-multiply shoots; (**d**) in vitro-rooted shoots; (**e**) hardened plantlets; and, (**f**) plant transferred to an experimental plot.

Among all of the tested concentrations of the commercial bleach and disinfection times, the highest yield of the process was achieved for the surface disinfection of *C. japonica* seeds with 50% solution, regardless of the treatment time; all the aseptic seeds developed into healthy seedlings. The new shoots were formed from shoot tip explants by axillary branching. The morphogenetic potential was dependent on the medium treatment (Figure 1). 

In the presented study, the highest number of *C. japonica* shoots (ca. 5.22) was obtained on Murashige & Skoog medium (MS) with 1.0 mg L^−1^ N6-benzyladenine (BA) + 1.0 mg L^−1^ indole-3-acetic acid (IAA) (Figure 1). However, similar responses were also observed for plant fragments that were cultivated on MS various plant regulator combinations (Table 1). 

It should be noticed that, regardless of the hormonal composition of the full-strength media (MS—Murashige & Skoog medium or WPM—Woody Plant Medium), the compensated number of shoots was received. However, those shoots that were placed on WPM were characterized by the lower content of chlorophyll and they were more elongated. Therefore, in the next stage of our research, this type of medium was not employed and only MS medium was adopted for shoot rooting. Other studies that were conducted on species from the Maloideae subfamily of the Rosaceae family also confirmed the observation; the composition of MS medium was more effective in *Malus domestica* L. shoot proliferation than the WPM [20]. In our study, the medium with reduced salt concentrations (½ MS, and ¾ MS) negatively affected the efficiency of the *C. japonica* shoot development process (Table 1). Panavas received different results [17]; the concentration of basal salts strongly affected shoot elongation and proliferation—the best shoot development of this species was achieved on ¾ MS. In the first experiment on *C. japonica* propagation, the highest number of shoots (ca. 2.6) was achieved in the presence of 2.0 mg L^−1^ BA, but a high concentration of BA caused a high level of vitrification [17]. In the studies of Bach et al. [18], the rate of proliferation for *C. japonica* shoot tip explants that were cultivated on MS medium was 2.1 and one nodal shoot explant was 1.5. The highest number of shoots was obtained on MS media with 0.9 or 1.8 mg L^−1^ BA. As the authors indicated, BA was more favorable in the process of micropropagation than 2iP [18]. On the other hand, Kauppinien [21] noticed that the shoots of this species do not proliferate easily, and for this reason seven MS media that were modified by varying concentrations of iron chelate, macronutrients, and BA (0.5–2.0 mg L^−1^) were tested. However, the response to different proliferation media was genotype-dependent [19]. In the following years, the author detailed his studies; the proliferation rate for *C. japonica* was 1.8, 2.2, and 1.4, depending on the genotype, and it increased for shoots that were cultured under low irradiation level to 4.1, 3.9, and 1.8 [19]. Furthermore, in vitro induction of polyploid plants of *C. japonica*, as a method to induce variation and enhance secondary metabolites production, was conducted via the maintenance of aseptic microshoots that were treated with cytostatic on ¾ MS medium (later cultivation on Nitsch-Nitsch medium) with BA [22]. In the studies of *M. domestica*, BA had a positive effect on multiple shoot formation [23]. According to the literature, benzyladenine has stimulated both axillary and adventitious shoot formation in plants from Rosaceae [24,25]. In our studies (BA presence in the medium), the shoot number of *C. japonica* did not decline over a few passages (data not shown), which supports the view that the axillary shoots can be subcultured without any adverse effect [25]. 

In vitro rooting is a crucial point in micropropagation, especially for woody plant species, including fruit trees of the Rosaceae family [26]. The rooting of shoots turned out to be the most difficult stage of *C. japonica* micropropagation as roots spontaneously formed on different variants of the culture media without a clear trend (Figure 1, Table 2). 

The salt composition of medium (½ MS, ¾ MS, and MS) and the supplementation with sucrose at different concentrations (15, 30, 60 g L^−1^) did not favor the induction of larger number of roots. The highest number of main roots was obtained from shoots that were cultured on MS + 15 g L^−1^ Suc + 1.0 mg L^−1^ IAA (3.00), MS + 60 g L^−1^ Suc + 1.0 mg L^−1^ IAA (2.50), and ½ MS + 30 g L^−1^ Suc without auxin (2.42) and ¾ MS + 30 g L^−1^ Suc without auxin (2.29). It can be seen in all cases that the addition of IAA affected the percentage of root induction and their number (Table 2). Other authors also confirmed the difficulty in *C. japonica* rooting. The efficiency of rooting was very low; just one root per 10 shoots was observed on MS medium with the presence of indole-butyric acid (IBA) (1.0 mg L^−1^) [17], IBA (0.1 mg L^−1^), or α-naphthaleneacetic acid (NAA) (0.09 mg L^−1^, 0.47 mg L^−1^ or 0.93 mg L^−1^) and three roots per 10 shoots on MS with IBA at a concentration of 0.5 mg L^−1^ [18,19]. It was also stated that the shoots of *C. japonica* do not root easily, and for this reason six rooting treatments were tested. Immediate planting of shoots resulted in poor rooting (also just one root per 10 shoots was observed) when compared to planting shoots that were pretreated with IBA (2.0 mg L^−1^) for two weeks (68%); this may suggest that the absence of BA during the pretreatment time improved rooting [19]. Shoots that were cultured on MS medium without phytohormones, after soaking in 250 mg L^−1^ IBA solution for 2 h, rooted easier −25% [17], ca. 45–90% [21]. On the other hand, the selected shoots of *C. japonica* were well rooted on auxin-free MS medium, but before acclimatization they were immersed in 300 mg L^−1^ IBA for 2 h [22]. High frequency rooting for *Malus domestica* L. was observed on ½ MS with 3.0 mg L^−1^ IBA [23] and on ½ MS with 1.5 mg L^−1^ IBA [20]. 

The acclimatization of micropropagated plantlets was high, namely 85% (Figure 1). Better results were obtained in previous studies, in which *C. japonica* in vitro-derived plantlets were acclimatized with a 98% survival rate [18]. In turn, the worst result was obtained for *M. domestica*, with the survival frequency of 60% of plants with well-developed roots [23].

**Figure 2:**
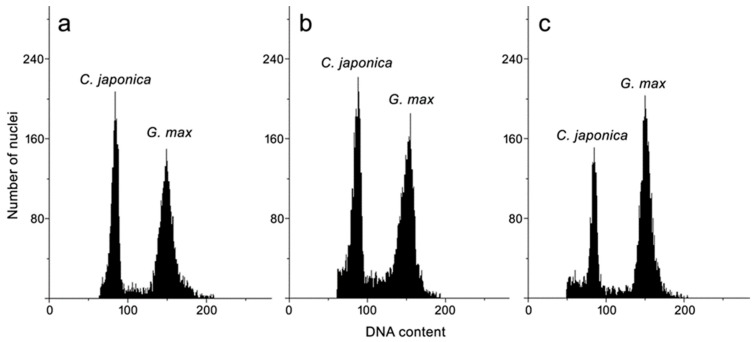
Selected histograms of nuclear DNA content obtained after flow cytometric analysis of the PI-stained nuclei isolated simultaneously from the leaves of *Glycine max* (internal standard) and *Chaenomeles japonica*: (**a**) leaves of field-grown plant; (**b**) leaves of aseptic seedlings; and, (**c**) leaves from in vitro-propagated plantlets (shoot culture).

The flow cytometry method has been used for many medicinal plants that were obtained in vitro to verify their genetic stability [27,28,29,30,31]. In this study, the cytometrically established genome size of *C. japonica* was about 1.4 pg/2C (Table 3, Figure 2). 

It provided evidence that in vitro-propagated plantlets maintained the same content of nuclear DNA as seedlings and intact plants; thus, the in vitro method of propagation via axillary buds proliferation that is proposed here can be recommended for production of true-to-type plantlets of *C. japonica*. The genome size of other Chaenomeles has been established previously just for one species, namely *C. speciosa* (1.2 pg/2C) [32]. This is the first report on the genome size of *C. japonica*. 

**Figure 3:**
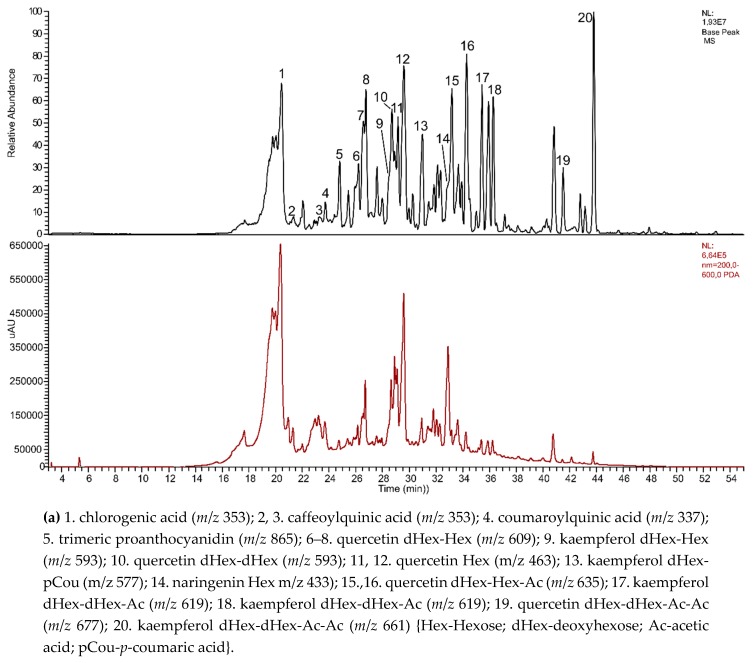

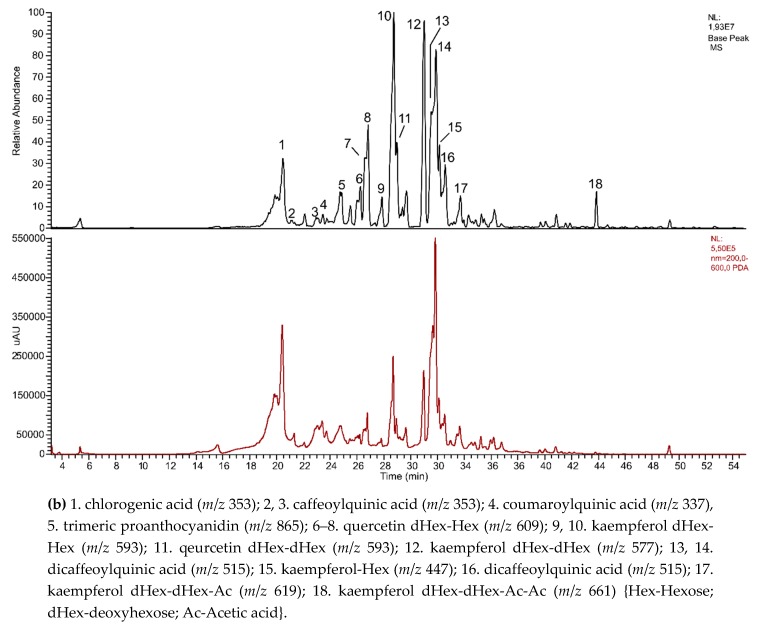
HPLC-MS and UV chromatograms (200–600 nm) of methanolic extracts from *Chaenomeles japonica* (**a**) leaves of field-grown plant and (**b**) micropropagated plantlets (shoot culture).

The qualitative HPLC analyses confirmed the presence of phenolic acids, mainly isomers of chlorogenic acid as well as flavonoids, mostly quercetin, and kaempferol derivatives, in the tested extracts from leaves of micropropagated plantlets (shoots cultures) and a field-grown plant of *C. japonica*. The results showed that the number of compounds was higher in the extract from the leaves of ground plants (Figure 3). 

Generally, leaves of naturally grown plants have a higher concentration of polyphenols, including phenolic acids and flavonoids, so similar activity in the antioxidant test may confuse (Table 4). 

The mean scavenging activity for the DPPH was IC_50_ = 0.09 mg mL^−1^ for leaves from in vitro-propagated plantlets and IC_50_ = 0.04 mg mL^−1^ for leaves from field-grown plants (Table 5). The results of antioxidant activity assessed with the CUPRAC and FRAP assays for the extracts of leaves from shoot cultures (IC_50_ = 1.25 mg mL^−1^ and 0.31 mg mL^−1^, respectively) and from field-grown plants (IC_50_ = 1.25 mg mL^−1^ and 0.33 mg mL^−1^, respectively) were similar for both of them (Table 5). 

The fruit of *C. japonica*, which is used in the food industry and well phytochemically characterized in the literature, being used as the reference, had a lower content of polyphenols but higher antioxidant activities measured with various methods. Chlorogenic acid and its isomers with documented antioxidant activity and the proven biological effects of many raw plant materials dominated in the extracts of *C. japonica* [33,34]. Moreover, the extracts of *C. japonica* are rich in flavonoids that exhibit high antioxidant activity [35]. 

**Figure 4:**
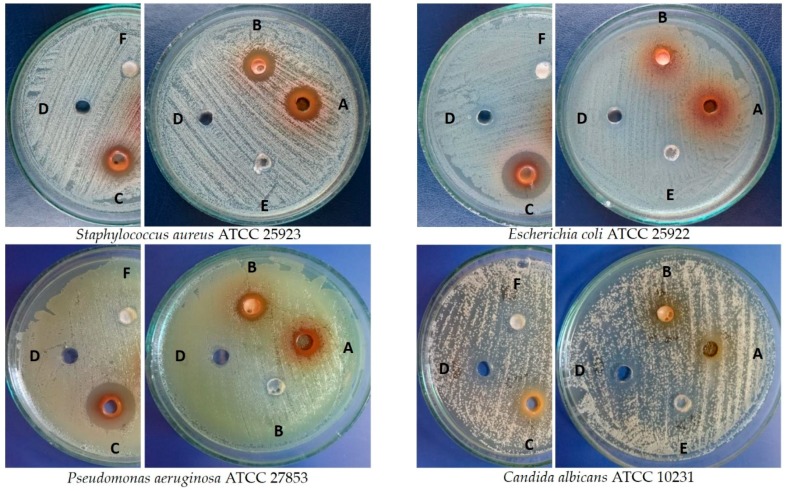
Inhibition effect of *Chaenomeles japonica* extracts on microbes A-leaves of micropropagated plantlets (shoot culture); B—leaves of field-grown plant; C—fruit; D—DMSO (dimethyl sulfoxide); E—ursolic acid; and, F—oleanolic acid.

The tested extracts of *C. japonica* showed various degrees of activity against the bacterial and yeast standard strains employing the agar well diffusion method (Figure 4, Table 6). 

The fruit extract that was used as a reference material in this study (rich in many metabolites—see the Introduction part) exhibited maximum activity against *E. coli* ATCC 25922 and *P. aeruginosa* ATCC 27853, as well as moderate activity against *S. aureus* ATCC 25923 and *C. albicans* ATCC 10231 (the mean zones of inhibition were 20.0 ± 0.0 mm, 19.3 ± 0.6, 14 ± 0.0 mm, and 11.3 ± 0.6 mm, respectively), whereas the extract from the leaves of field-grown plants was the most active against *S. aureus* ATCC 25923 (Figure 4, Table 6). Moderate activity of the tested extracts was found against *E. coli* ATCC 25922 (the extracts from leaves of field-grown plants) and *P. aeruginosa* ATCC 27853 (the extract from shoot culture of in vitro-propagated plantlets and from leaves of field-grown plants). No influence of the solvent that was used for preparation of the extracts stock solutions on the obtained results was observed for *S. aureus* and *E. coli*, whereas the growth of *C. albicans* and *P. aeruginosa* was slightly inhibited. Amikacin and fluconazole, being applied as the reference substances, significantly inhibited the growth of the tested microorganisms, whereas the oleanolic acid and ursolic acid slightly influenced the growth of *C. albicans* (the growth of very small colonies around the wells was observed) (Table 6). The extracts of the fruits and the essential oil from *C. speciosa* (Sweet) Nakai, which is another species from Chaenomeles, exhibited antibacterial activity [12]. Moreover, the extracts from the fruits of *Cydonia oblonga* Mill. were active in inhibiting the bacteria growth. The authors suggested the crucial role of chlorogenic acid acting in synergism with other compounds of the extract [36]. 

## 3. Materials and Methods 

### 3.1. Plant Material and Surface Disinfection

The voucher specimens of *C. japonica*, under the number 1526/2016, were deposited in the Institute of Natural Fibers and Medicinal Plants in Poznan, Poland. 

The mature fruits of *C. japonica* were collected from a 45-year-old shrub growing in the garden in Poznan, Poland (52°21′55″ N 17°00′12″ E), in 2013. The ripe fruits were picked when the seeds turned brown. The seeds were isolated from the fresh fruits and the surface was then decontaminated applying 70% (*v*/*v*) ethanol for 30 s and 20% (*v*/*v*) commercial bleach (sodium hypochloride) with Tween 80 for 10 min. They were rinsed five times and then put on lignin in thermostat (26 °C) for 24 h to swell the seeds. The accurate disinfection step was 50% (*v*/*v*) commercial bleach for various periods (10, 15, and 20 min). They were rinsed five times in sterilized water and transferred to MS [37] or WPM [38] medium to obtain the aseptic seedlings. 

Each medium consisted of MS or WPM salts, which were solidified with 0.8% (*w*/*v*) agar (Sigma Aldrich, Saint Louis, MO, USA), supplemented with 15, 30, or 60 g L^−1^ (*w*/*v*) sucrose and plant growth regulators (PGRs) at various concentrations (Table 1 and Table 2). The PGRs were purchased from Sigma-Aldrich (Saint Louis, MO, USA). The pH was adjusted to 5.8 using 1 M KOH before the media were autoclaved (121 °C, 105 kPa, 20 min). The cultures were maintained in the growth room (16/8 h photoperiod, 55 μmol m^−2^·s^−1^ light, temperature 21 °C). 

### 3.2. Establishment of Shoot Cultures and Effect of PGRs on Shoot Multiplication 

After four weeks, the in vitro-germinated seedlings were the sources of explants for proliferation of new shoots. Single shoots were placed in (i) WPM, (ii) MS, and (iii) ½ MS (reduced concentration of macro- and micronutrients). The media were enriched with N6-benzyladenine (BA), indole-3-acetic acid (IAA), α-naphthaleneacetic acid (NAA) at various concentrations, or with PGRs (Table 1). The explants were transferred to the freshly prepared medium every six weeks of subculture until the 12th generation. The multiplication process was replicated six/seven times with at least 30 explants/treatment. 

### 3.3. Rooting of Shoots and Plantlet Acclimatization

Healthy shoots of about 2.5–4 cm long were separated from the in vitro-multiplied shoots and the transferred to ½ MS, ¾ MS, and MS media, alone or with one of the three auxins, namely indole-3-acetic acid (IAA), indole-butyric acid (IBA) or α-naphthaleneacetic acid (NAA), and sucrose (Suc) at different concentrations (15, 30, or 60 g L^−1^) for root induction and growth (Table 2). The experiment was replicated two/three times with at least 10 explants per treatment. The healthy plants were subsequently transplanted to black plastic pots (90 mm in diameter) containing the autoclaved mixture of soil, sand, and perlite (4:2:1 *v*/*v*/*v*), and were then covered with transparent containers (optimum humidity) for 14 days (24 ± 2 °C, natural light). Subsequently, the plants were transferred to the plot. After the acclimatization of plantlets, the survival rate was calculated and phenotypic observations were made. 

### 3.4. Genome Size Estimation

Nuclear DNA content estimation of the leaves of a young field-grown plant, in vitro-growing seedling, and in vitro-propagated plantlets was carried out according to the previously described method [29,30,31,39]. A nuclei isolation buffer [40] with 1% (*v*/*v*) polyvinylpyrrolidone, propidium iodide (50 µg cm^−3^), and ribonuclease A (50 µg cm^−3^) was applied. *Glycine max* ‘Polanka’ (2.50 pg/2C) [41] was used as an internal standard. The analyses were replicated five times. The coefficient of variation of the G0/G1 peak of *C. japonica* ranged from 5.02 to 6.96%. 

### 3.5. Phytochemical Screening of Polyphenols

The samples of plant material-leaves from in vitro-propagated plantlets (shoot culture) and field-grown plants (100 mg) were extracted with 80% (*v*/*v*) methanol. The ASE 200 system (Dionex, Sunnyvale, CA, USA) was used. Extraction (three cycles) was performed with 80% MeOH at 1500 psi solvent pressure, with the cell temperature of 40 °C, and the flush of 150%. The extracts (25 mL) were collected into glass vials. The crude extracts were carefully evaporated to dryness, reconstituted in 5% (*v*/*v*) MeOH (1.0 mL), and then subjected to solid phase extraction on (a) SepPak (Waters, Miliford, MA, USA) that was equilibrated with 5% MeOH. The analytes were eluted with 80% MeOH, carefully evaporated to dryness, and reconstituted with 80% (*v*/*v*) MeOH (1.0 mL). The samples were stored in −20 °C. The analytes were centrifuged at 23,000× *g* for 15 min, immediately before the analysis. HPLC-ESI-ITMS system with the Thermo Surveyor HPLC and the LCQ Advantage Max ion trap mass spectrometer (Thermo Scientific, San Jose, CA, USA) was applied. The HPLC separations were performed on Waters XBridge BEH C18 reversed-phase column (2.5 µm, 3 mm × 150 mm) (Waters, Milford, MA, USA). The analytes were separated while using a 95-min long linear gradient from 2 to 75% of a mobile phase B (acetonitrile with 0.1% (*v*/*v*) formic acid) in a mobile phase A (HPLC-grade water with 0.1% (*v*/*v*) formic acid). After each separation, the column was flushed with 100% of phase B for 6 min and then equilibrated to initial gradient conditions over 12 min (flow rate—0.3 mL min^−1^, column temperature—50 °C). The flow from the chromatographic system was introduced into the ESI ion source operating in the negative ion mode with capillary voltage—17 V, spray voltage—3.9 kV, tube lens offset—60 V, capillary temperature—255 °C, sheath gas (N2) flow rate—70 (arbitrary units), and auxiliary gas (N2) flow rate 10 (arbitrary units). The scan range was *m*/*z* 100–2000 and the maximum injection time was 150 ms with three microscans. The two scan events were arranged to run sequentially. The first event was a full-scan mass spectrum to acquire data on anions in the designated scan range. The latter event, which was triggered if the abundance of an ion in the first scan event exceeded 1.5 × 105 counts, was MS/MS fragmentation at the normalized collision energy of 35%, which was performed on the most prominent of acquired [M − H]^−^ ions. 

### 3.6. Determination of Total Phenolic, Phenolic Acid, and Flavonoid Contents

The samples of the lyophilized and powdered biomass were extracted three times with a matched quantity of 80% (*v*/*v*) ethanol for 60 min at the boiling point temperature of the extractive mixture under reflux. The extracts were evaporated to dryness under reduced pressure. 

The content of phenolics in the ethanol-water extracts (80%, *v*/*v*) was spectrophotometrically determined while employing a [42] modified method with the Folin–Ciocalteu reagent. Briefly, 0.05 mL of the tested extract was mixed with 3.7 mL of distilled water and 0.25 mL of the Folin–Ciocalteu reagent; the mixture was vigorously shaken and 20% (*w*/*v*) sodium dicarbonate solution (1.0 mL) was added after 1 min. Subsequently, the samples were incubated in the dark for 30 min at room temperature. The absorbance was measured at 760 nm. The concentration of the total phenolic compounds in the extract was expressed as mg of gallic acid equivalent per gram of dry plant material weight from a calibration curve of gallic acid (y = 9.8399x + 0.0289, R^2^ = 9993) at a concentration range (20–80 µg 10 mL^−1^). The values were expressed as the mean of six replications ± SD.

The content of phenolic acids in the ethanol-water extracts (80%, *v*/*v*) was determined while applying a spectrophotometric method with Arnov’s reagent (10.0 g Na_2_MoO_4_, 10.0 g NaNO_2_ in 100.0 mL water) defined in Polish Pharmacopeia (VI edition, 2002) [43]. The sample (1.0 mL) was shaken with 5.0 mL of water and then with HCl (18 g L^−1^), Arnov’s reagent, and NaOH (40 g L^−1^); with each of them being added in the volume of 1.0 mL. The volume was completed to 10.0 mL with distilled water. The absorbance was measured at 490 nm. The total content of the phenolic acids was presented as mg of caffeic acid (40–200 µg 10 mL^−1^) per gram of dry plant material weight from a calibration curve of caffeic acid (y = 11.382x + 0.0746, R^2^ = 0.9834) at a concentration range (40–300 µg 10 mL^−1^). The values were expressed as the mean of five replications ± SD. 

The content of flavonoids in the ethanol-water extracts (80%, *v*/*v*) was determined according to Meda et al. (2005) [44]. Briefly, equal volumes of 2% AlCl_3_ in methanol (0.7 mL) and the extract (0.7 mL) were combined and then incubated for 10 min at room temperature. The absorbance was read at 415 nm while using water and methanol without AlCl_3_ as a blank. The total concentration of the flavonoid content was expressed as mg of quercetin per gram of dry plant material weight from a calibration curve of quercetin (y = 0.046x – 0.0199, R^2^ = 0.9991) at a concentration range (3.125–25 µg mL^−1^). The values were expressed as the mean of six replications ± SD.

### 3.7. Determination of Antioxidant Activity Using DPPH, CUPRAC, and FRAP Assays

The free radical scavenging capacity of the ethanol-water extracts (80%, *v*/*v*) was evaluated by the DPPH analysis, as described by Annegowda et al. [45]. Briefly, 0.05 mL of the tested samples dissolved in DMSO (Avantor Performance Materials Poland S.A., Gliwice, Poland) was added to 1.45 mL of the DPPH (Sigma-Aldrich, St. Louis, MO, USA) solution in methanol (0.1 Mm). The sample was shaken and then incubated at room temperature in the dark for 30 min. The absorbance was read at 517 nm against a blank (1.45 mL of methanol + 0.05 mL of DMSO). The negative control was a mixture of 1.45 mL of the DPPH reagent and 0.05 mL of DMSO; vitamin C (Avantor Performance Materials Poland S.A., Gliwice, Poland) was used as a positive control. The concentration of the DPPH radicals was calculated using the following equation of the concentration of the DPPH radicals: DPPH radical scavenging (%) = (A0 − A1/A0) × 100, where A0 is the absorbance of the negative control and A1 is the absorbance of the reaction mixture or standards. The IC_50_ values of the antioxidant were calculated as the concentration that is required to inhibit activity of the DPPH radicals by 50%. The values were expressed as the mean of six replications ± SD.

The antioxidant activity of the ethanol-water extracts (80%, *v*/*v*) was determined by the CUPRAC assay [46]. The studies were conducted at a constant temperature of 24 ± 1 °C. Briefly, 0.4 mL of the tested samples (dissolved in water at the concentrations of 100 µg, 200 µg, 400 µg, 600 µg, and 800 µg of the material/1.0 mL) were mixed with 1.0 mL of CuCl_2_ solution (10 mM, Avantor Performance Materials Poland S.A., Gliwice, Poland), 1.0 mL of ethanolic solution of neocuproine (Sigma-Aldrich, St. Louis, MO, USA), 1.0 mL ammonium acetate buffer (pH = 7.0), and 0.6 mL of water. The absorbance was measured at λ = 450 nm, after 30 min of incubation. Based on the calibration curve (y = 0.004x + 0.1183, y = 0.004x + 0.0871, y = 0.004x + 0.0951, y = 0.3663x + 0.0063 for leaves from shoot culture, a field-grown plant, a fruit, and buthylated hydroanisole (BHA), respectively), the IC_50_ for the test and the standard samples was calculated. The values were expressed as the mean of three replications ± SD. BHA (buthylated hydroanisole, Fluka, Sigma-Aldrich, Steinheim, Germany) was used as a standard (0.2–2.0 µg mL^−1^).

The FRAP method is based on the reduction of ferric (Fe^3+^) to ferrous (Fe^2+^) ions at low pH. The method described by Tiveron and others [47], with minor modifications, was used to effectuate the analysis [47]. Briefly, 0.5 mL of prepared extract was added to 1.5 mL of the FRAP solution. After 30 min of incubation at 37 °C the absorbance was measured at λ = 593 nm with a spectrophotometer (UV/VIS Lambda 35 Elmer-Perkin). Based on the calibration curve (y = 0.0016x + 0.0512, y = 0.0015x + 0.3802, y = 0.018x + 0.1049, y = 0.3242x − 0.0188 for leaves from shoot culture, a field-grown plant, a fruit and BHA, respectively), the IC_50_ for the test and standard samples was calculated BHA (Fluka, Sigma-Aldrich, Steinheim, Germany) was used as a standard (0.2–2.0 µg mL^−1^).

### 3.8. Antimicrobial Assay

The extracted and evaporated biomass was suspended in DMSO (Avantor Performance Materials Poland S.A., Poland) to yield the concentration of 100 mg mL^−1^. 

The strains of *Staphylococcus aureus* (ATCC 25923), *Escherichia coli* (ATCC 25922), *Pseudomonas aeruginosa* (ATCC 27853), and *Candida albicans* (ATCC 10231) originated from the American Type Culture Collection. The bacteria strains were cultured in brain-heart infusion broth (BHI; OXOID, UK), while the *C. albicans* were cultured in Sabouraud dextrose broth (SDB; OXOID, UK) at 36 °C ± 1 °C for 20 h. The organisms were harvested by centrifugation (3000 rpm for 15 min), re-suspended in 10 mM PBS, pH 7.0 (Sigma-Aldrich, Saint Louis, MO, USA), and then diluted in a suitable medium.

The antimicrobial activity of the extracts under investigation was determined by the agar well diffusion method. Mueller-Hinton agar (MHA; OXOID, UK) and Sabouraud dextrose agar (SDA; OXOID, UK) plates were inoculated with the microbe suspension (each containing about 10^5–^10^6^ CFU mL^−1^). Subsequently, 6 mm wells were punched into the agar plates. Next, 50 µL aliquots of each tested biomass extract (100 mg mL^−1^) were dispensed into each cylinder. DMSO was used for each microbial strain as a control. Amikacin and fluconazole were used as standard antimicrobials. The plates were incubated for 18h at 36 °C ± 1 °C. Antimicrobial activity was evaluated by measuring the zones of inhibition [mm]. The growth of microbes was measured. All of the tests were performed in triplicates. 

### 3.9. Statistical Analysis

The data were analysed using a one-way analysis of variance (ANOVA). Duncan’s multiple range post hoc test was used for the statistical significance declaration (*p*-value of 0.05). All the analyses were conducted using STATISTICA v. 13 (StatSoft2015, Kraków, Poland) and the data were presented as mean values. 

## 4. Conclusions

In conclusion, our research confirmed the regeneration potential of genetically-stable plants of *C. japonica* under in vitro conditions, the ability of in vitro-derived plantlets to produce polyphenolics as those that are present in wild plants as well as the antioxidant potential of the leaf extract. Although the activity of the extracts from fruits has been known in Chinese traditional medicine and it has been confirmed in numerous research papers, the extracts from leaves of *C. japonica* are also worth paying attention to due to the presence of the bioactive compounds with biological activity.

## Figures and Tables

**Table 1:** Effect of Murashige & Skoog medium (MS) medium composition and growth regulators on shoot multiplication from shoot tip explants of *Chaenomeles japonica* after six weeks of in vitro culture.

Medium	Cytokinin [mg L^−1^]	Auxin [mg L^−1^]	Shoot Induction [%]	Shoot No./Explant ± SE	Shoot Length [cm] ± SE
	**N6-benzyladenine (BA)**	**Indole-3-acetic acid (IAA)**			
WPM	-	-	100	4.17 ± 0.10 ^ab^	1.55 ± 0.06 ^bd^
WPM	1.0	0.1	100	4.14 ± 0.12 ^ab^	1.30 ± 0.07 ^bd^
½ MS	-	-	100	1.03 ± 0.04 ^c^	1.25 ± 0.01 ^bd^
MS	-	-	100	4.62 ± 0.21 ^ab^	2.40 ± 0.17 ^a^
MS	2.0	-	100	4.38 ± 0.12 ^ab^	0.82 ± 0.03 ^c^
MS	1.0	-	100	4,37 ± 0.20 ^ab^	0.92 ± 0.04 ^cd^
MS	1.0	1.0	100	4.70 ± 0.05 ^ab^	1.00 ± 0.04 ^cd^
MS	1.0	0.1	100	5.22 ± 0.12 ^a^	2.38 ± 0.14 ^a^
	**BA**	**α-naphthaleneacetic acid (NAA)**			
MS	1.0	1.0	100	4.33 ± 0.13 ^ab^	0.95 ± 0.04 ^cd^
MS	1.0	0.1	100	4.01 ± 0.16 ^b^	1.16 ± 0.10 ^bcd^

Mean values within a column with the same letter are not significantly different at *p* = 0.05 using Duncan’s test.

**Table 2:** Effect of medium salts, sugar concentration and auxins on rooting of *Chaenomeles japonica* shoots after six weeks of in vitro culture.

Medium			Root Induction [%]	Root No./Explant ± SE	Root Length (cm) ± SE
Basal Nutrition	Sucrose [g L^−1^]	Auxin [mg L^−1^]			
½ MS	30	-	95	2.42 ± 0.19 ^ab^	6.71 ± 0.58 ^ab^
½ MS	30	IAA 1.0	0	0.00 ± 0.00	0.00 ± 0.00
½ MS	30	IBA 1.0	0	0.00 ± 0.00	0.00 ± 0.00
½ MS	30	NAA 1.0	0	0.00 ± 0.00	0.00 ± 0.00
¾ MS	30	-	85	2.29 ± 0.22 ^abc^	8.35 ± 1.24 ^a^
¾ MS	30	IAA 1.0	0	0.00 ± 0.00	0.00 ± 0.00
¾ MS	30	IBA 1.0	0	0.00 ± 0.00	0.00 ± 0.00
¾ MS	30	NAA 1.0	0	0.00 ± 0.00	0.00 ± 0.00
MS	15	-	0	0.00 ± 0.00	0.00 ± 0.00
MS	30	-	30	1.33 ± 0.21 ^c^	2.75 ± 0.44 ^c^
MS	60	-	20	1.25 ± 0.25 ^c^	1.90 ± 0.33 ^c^
MS	15	IAA 1.0	50	3.00 ± 0.30 ^a^	2.09 ± 0.32 ^c^
MS	30	IAA 1.0	45	1.33 ± 0.16 ^c^	2.12 ± 0.25 ^c^
MS	60	IAA 1.0	70	2.50 ± 0.48 ^ab^	3.95 ± 0.54 ^bc^
MS	15	IBA 1.0	0	0.00 ± 0.00	0.00 ± 0.00
MS	30	IBA 1.0	81	2.05 ± 0.20 ^abc^	3.17 ± 0.57 ^c^
MS	60	IBA 1.0	0	0.00 ± 0.00	0.00 ± 0.00
MS	15	NAA 1.0	0	0.00 ± 0.00	0.00 ± 0.00
MS	30	NAA 1.0	40	1.50 ± 0.19 ^bc^	1.88 ± 0.33 ^c^
MS	60	NAA 1.0	0	0.00 ± 0.00	0.00 ± 0.00

Mean values within a column with the same letter are not significantly different at *p* = 0.05 using Duncan’s test.

**Table 3:** Nuclear DNA content in leaves of *Chaenomeles japonica* field-grown plant, seedling, and in vitro-propagated plantlets.

Plant Material	DNA Content (pg/2C ± SD)
Leaf from field-grown plant	1.43 ± 0.02 ^ns^
Leaf of seedling	1.41 ± 0.02
Leaf of shoot culture	1.41 ± 0.02

ns—no significant differences at *p* = 0.05 (Duncan’s test).

**Table 4:** Total polyphenols, phenolic acids, and flavonoids in ethanolic extracts from leaves of in vitro-propagated plantlets, field-grown plant, and fruits of *Chaenomeles japonica*.

	Content of Selected Groups of Compounds in Extracts [± SD]		
Total	Leaves from Shoot Culture	Leaves from Field-Grown Plant	Fruits
**POLYPHENOLS** [mg GAE g^−1^]	57.42 ± 1.5	130.83 ± 2.5	26.16 ± 0.80
**PHENOLIC ACIDS** [mg CA g^−1^]	23.62 ± 0.23	64.81 ± 0.76	18.01 ± 0.37
**FLAVONOIDS** [mg QE g^−1^]	30.51 ± 0.45	77.45 ± 2.5	0.33 ± 0.00

R-reference compound.

**Table 5:** Antioxidant effect of ethanolic extracts from leaves of in vitro-propagated plantlets, field-grown plant, and fruits of *Chaenomeles japonica*.

Method	Extracts IC_50_ (mg mL^−1^)			Standard	IC_50_ (µg mL^−1^)
	Leaves from Shoot Culture	Leaves from Field-Grown Plant	Fruits		
**DPPH**	0.09	0.04	0.14	-	-
**CUPRAC**	1.25	1.25	0.99	BHA	1.35
**FRAP**	0.31	0.33	0.27	BHA	1.60

BHA—buthylated hydroanisole.

**Table 6:** Antimicrobial activity of *Chaenomeles japonica* extracts.

Microbes	Zone of Inhibition (mm) ± SD against Microbial Strains							
	Leaves from Shoot Culture [5 mg]	Leaves from Field-Grown Plant [5 mg]	Fruits [5 mg]	DMSO	AN ^1^ [25 µg]	FZ ^2^ [50 µg]	UA ^3^ [50 µg]	OA ^4^ [50 µg]
***Staphylococcus aureus*** ATCC 25923	**14.3** ± 0.6	**14.0** ± 0.0	**14.0** ± 0.0	**6.0** ± 0.0	**24.7** ± 0.6	NT	**6.0** ± 0.0	**6.0** ± 0.0
***Escherichia coli*** ATCC 25922	**7.0** ± 1.0	**7.3** ± 0.6	**20.0** ± 0.0	**6.0** ± 0.0	**26.7** ± 0.6	NT	**6.0** ± 0.0	**6.0** ± 0.0
***Pseudomonas aeruginosa*** ATCC 27853	**11.0** ± 0.0	**10.7** ± 0.6	**19.3** ± 0.6	**6.3** ± 0.6	**25.3** ± 0.6	NT	**6.0** ± 0.0	**6.0** ± 0.0
***Candida albicans*** ATCC 10231	**8.3** ± 1.2	**7.3** ± 0.6	**11.3** ± 0.6	**6.7** ± 0.6	NT	**25.0** ± 0.0	**6.0** ± 0.0	**6.0** ± 0.0

^1^ AN—amikacin, ^2^ FZ—fluconazole, ^3^ UA—ursolic acid, ^4^ OA—oleanolic acid, NT—not tested.
